# Association between metabolic score for insulin resistance and stroke: a nationally representative cross-sectional study from NHANES 2007–2018

**DOI:** 10.3389/fneur.2024.1478884

**Published:** 2025-01-03

**Authors:** Lingtian Weng, Yuqiu Lu, Hanning Song, Jiayi Xu, Xuhong Jiang

**Affiliations:** ^1^The First School of Clinical Medicine, Zhejiang Chinese Medical University, Hangzhou, Zhejiang Province, China; ^2^The Second School of Clinical Medicine, Zhejiang Chinese Medical University, Hangzhou, Zhejiang Province, China; ^3^General Office, Office of the President, Development Planning Department, Zhejiang Chinese Medical University, Hangzhou, Zhejiang Province, China

**Keywords:** stroke, metabolic syndrome, insulin resistance, NHANES, cross-sectional study

## Abstract

**Background:**

Stroke is a significant cerebrovascular disease and remains one of the leading causes of death and disability worldwide. Insulin resistance has been strongly linked to the incidence of stroke. Employing characteristics of metabolic syndrome, the Metabolic Score for Insulin Resistance (METS-IR) accurately measures insulin resistance. Nonetheless, the relationship between METS-IR and stroke risk is not well-established.

**Methods:**

We analyzed data from the National Health and Nutrition Examination Survey (NHANES) covering the years 2007–2018. Participants providing complete METS-IR data and self-reported stroke information were included in the study. We utilized weighted multivariate regression to explore the relationship between METS-IR and stroke, performing subgroup analyses as well.

**Results:**

A total of 14,794 participants were included, with an average METS-IR of 43.44 ± 12.68. The overall prevalence of self-reported stroke was 3.79%, with higher rates observed in upper METS-IR tertiles. An increase of one unit in METS-IR was associated with a 1% increase in stroke risk (OR = 1.01; 95% CI: 1.01–1.02). Interaction tests indicated no significant effects of gender, smoking status, alcohol consumption, hypertension, diabetes, physical activity, or serum cholesterol levels on this relationship. Notably, for participants younger than 60 years, the association was significantly stronger (OR = 1.02; 95% CI: 1.01–1.03), with a marked interaction (*p* = 0.0061).

**Conclusion:**

Our findings indicate a positive correlation between higher METS-IR and increased stroke risk. Early intervention targeting insulin resistance may be a viable preventive measure against stroke, particularly in individuals under 60 years of age.

## 1 Introduction

Stroke, a devastating cerebrovascular disease, is the world’s second leading cause of death and ranks among the top three causes of death and disability combined (1, 2). Global research indicates that each year approximately 14 million individuals suffer their first stroke, with 6 million dying and another 26 million living with its effects (3). Over recent decades, the incidence, mortality, and disability rates of stroke have steadily increased, especially in low- and middle-income countries (3–5). The economic impact of stroke, including direct medical costs and indirect losses due to decreased labor (6). The estimated yearly economic impact of stroke is substantial. Annually, stroke accounts for more than $721 billion globally, equivalent to 0.66% of the world’s Gross Domestic Product (GDP), placing significant strain on individuals, families, and societies (3, 7). The American Heart Association cites several risk factors for stroke, such as smoking, alcohol consumption, hypertension, Type 2 diabetes, obesity, high cholesterol, and an unhealthy diet (8). The prevalence of metabolic syndrome, which is closely linked to high-sugar, high-fat diets, is increasing (9). Metabolic syndrome is associated with cardiovascular disease and stroke (10).

Metabolic syndrome (MetS) comprises four main components: insulin resistance (IR), atherogenic dyslipidemia, visceral adiposity, and endothelial dysfunction (11, 12). Bello-Chavolla et al. (13) introduced the Metabolic Score for Insulin Resistance (METS-IR), a novel index that evaluates insulin sensitivity using fasting plasma glucose (FPG), high-density lipoprotein cholesterol (HDL-C), fasting triglycerides (TG), and body mass index (BMI). METS-IR has shown higher diagnostic sensitivity and reliability for assessing insulin resistance compared to other indices like the triglyceride glucose index (TyG) and the homeostasis model assessment for insulin resistance (HOMA-IR) (14). Studies suggest that METS-IR provides a more precise prediction of insulin sensitivity, surpassed only by the euglycemic-hyperinsulinemic clamp (EHC), the gold standard for insulin resistance measurement (15). Moreover, METS-IR has been extensively validated in predicting metabolic conditions such as visceral adiposity and the incidence of Type 2 diabetes (13, 16, 17).

Recent studies have underscored the predictive value of METS-IR concerning cardiovascular outcomes, including stroke. For instance, METS-IR has been positively linked with the risk of cardiovascular events in patients with hypertension and obstructive sleep apnea, making it a potent marker for forecasting adverse outcomes (18). Additionally, another study found a strong association between elevated METS-IR levels and an increased risk of ischemic stroke, indicating that patients with higher METS-IR scores are more prone to ischemic strokes than those with lower scores (19). Furthermore, a Mendelian randomization study provided evidence supporting a causal link between METS-IR and the risk of ischemic stroke, particularly pointing to its relevance in the small artery occlusion subtype of stroke (20). These findings highlight the critical role of monitoring METS-IR as part of a comprehensive strategy for predicting and managing stroke risk and other cardiovascular conditions.

This study aims to examine the association between METS-IR and stroke risk using data from the National Health and Nutrition Examination Survey (NHANES) from 2007 to 2018. We hypothesize that higher METS-IR levels correlate with an increased risk of stroke. By exploring this relationship, we aim to provide new insights into early interventions that could mitigate stroke risk by effectively managing insulin resistance.

## 2 Materials and methods

### 2.1 Sample selection

Data for this study were obtained from the National Health and Nutrition Examination Survey (NHANES), which utilizes a sophisticated multi-stage stratified probability sampling method to ensure a representative sample size (21). All NHANES research protocols received approval from the National Center for Health Statistics (NCHS) Research Ethics Review Board. Informed consent was obtained from all participants, or from parents and/or legal guardians for those under the age of 16.

**Figure 1:**
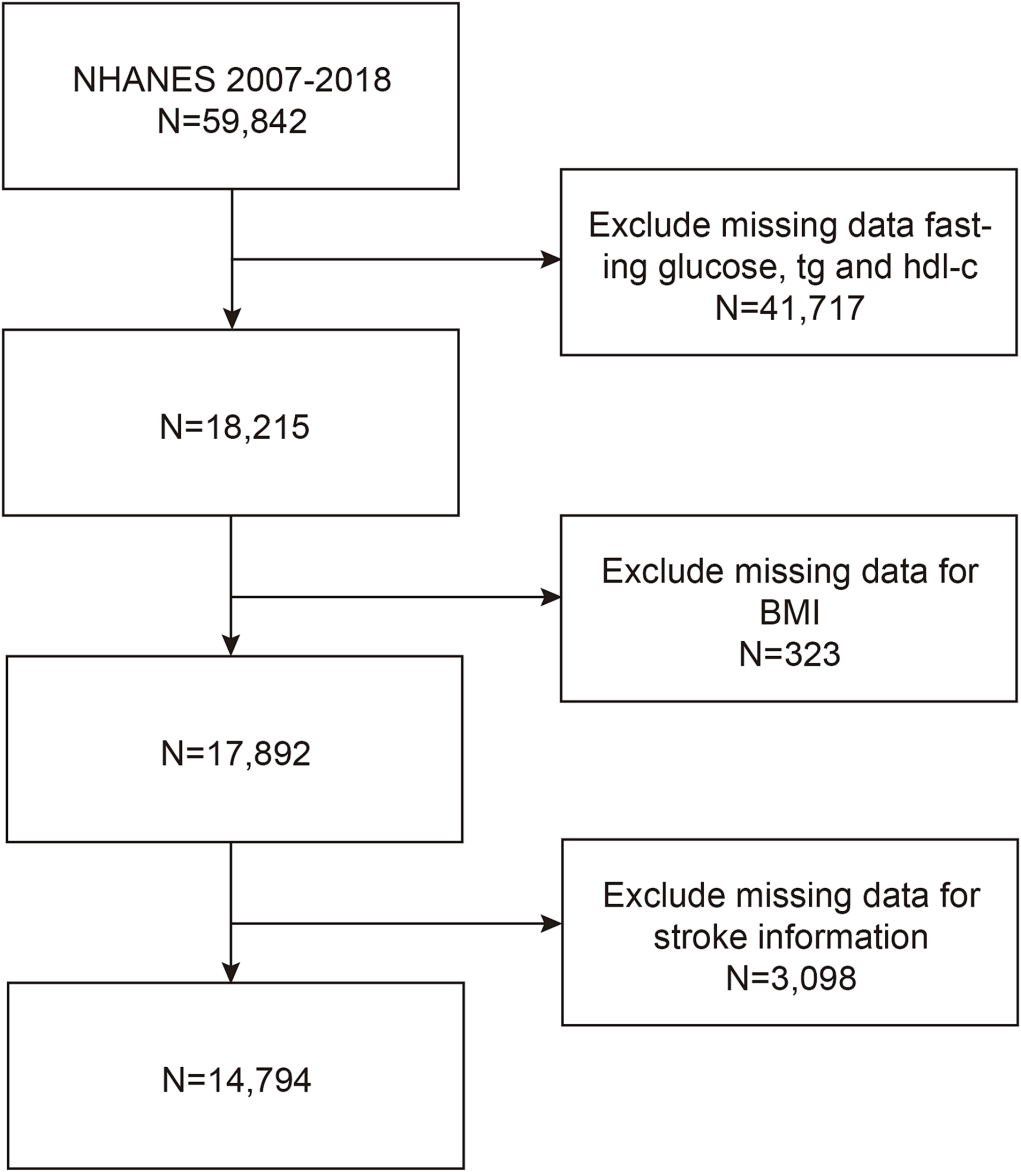
Participants screening flowchart.

For this study, we analyzed data from six NHANES cycles between 2007 and 2018. Initially, 59,842 participants were enrolled; however, 41,717 were excluded due to incomplete data on FPG, TG, HDL-C. An additional 323 participants were excluded because of incomplete data on BMI, and 3,098 were excluded due to unavailable stroke data. Ultimately, 14,794 participants were included in the study (Figure 1).

### 2.2 METS-IR and stroke

The METS-IR index was employed as an exposure variable and was derived using the following formula: $\mathrm{METS}-IR=\ln\left[\left(2\times FPG+TG\right)\times BMI\right]÷\ln\left(HDL-C\right)$ (13). After an 8.5-h fast, FPG and TG levels were measured with an automated biochemical analyzer. To calculate body mass index (BMI; kg/m^2^), we divided an individual’s weight (kg) by their height (m^2^).

Stroke was the outcome variable in this study. Based on the Medical Condition Questionnaire (MCQ) question no. 160f, individuals who answered “yes” were categorized as having had a stroke, while those who answered “no” were classified as non-stroke patients. The validity of self-reported strokes has been supported by previous studies (22, 23).

### 2.3 Covariates

In this study, covariates were selected based on prior research that identified risk factors associated with stroke (24, 25). These included age, gender, race, education levels, marital status, family poverty-to-income ratio, smoking status, alcohol consumption, hypertension, diabetes, physical activity levels, serum total cholesterol (mg/dL), and low-density lipoprotein cholesterol (LDL-C; mg/dL). Individuals were considered smokers if they had smoked at least 100 cigarettes in their lifetime. Alcohol consumption was defined by the question, “Had at least 12 alcoholic drinks in the past year.” Hypertension was determined if individuals self-reported the condition, used antihypertensive medication, or had a mean diastolic blood pressure ≥ 90 mmHg and/or a mean systolic blood pressure ≥ 140 mmHg. Diabetes criteria included the use of hypoglycemic medications, a diagnosis of diabetes, a 2-h plasma glucose level ≥ 200 mg/dL, a fasting plasma glucose level ≥ 126 mg/dL, or a hemoglobin A1c level ≥ 6.5%. Participants were categorized as engaging in vigorous physical activity if they participated in moderate-intensity sports for ≥10 min (26)(Wei et al., 2023).

### 2.4 Statistical methods

All statistical analyses adhered to the Centers for Disease Control and Prevention guidelines, considering complex multi-stage cluster surveys and using appropriate NHANES sample weights. Continuous variables are presented as means and standard deviations (SDs), while categorical variables are reported as proportions. Weighted Student’s t-tests were used for continuous data, and weighted Chi-Square tests were employed for categorical variables. To examine the characteristics of different METS-IR participants and their association with stroke incidence, METS-IR was categorized into tertiles. Multivariable logistic regression was used to explore the association between METS-IR and stroke incidence. Model 1 included no covariates. Model 2 was adjusted for gender, age, and race. Model 3, building on Model 2, also included adjustments for education level, marital status, family poverty-income ratio, smoking status, alcohol consumption, physical activity levels, serum cholesterol, and LDL cholesterol. Subgroup analyses were performed using stratified multivariable logistic regression models, stratifying by gender, age (<60 / ≥60 years), race, smoking status, alcohol consumption, hypertension, diabetes, physical activity levels, and serum cholesterol (<200 / ≥200 mg/dL). To manage the risk of Type I errors from multiple comparisons in subgroup analyses, we applied the false discovery rate (FDR) correction using the Benjamini-Hochberg procedure. This method was chosen to balance the control of false positives with the preservation of statistical power, particularly suitable for analyses involving multiple comparisons. Analyses were conducted using R version 4.1.3 and Empower software version 2.0. Statistical significance was set at a two-sided *p*-value <0.05.

## 3 Results

### 3.1 Baseline characteristics stratified by METS-IR tertiles

The present study included 14,794 participants, with an average age of 50.01 ± 17.61 years; 48.26% were male, and 51.74% were female. The prevalence of stroke among participants was 3.79%, increasing with each METS-IR tertile: 3.18% in tertile 1, 3.95% in tertile 2, and 4.24% in tertile 3 (*p* < 0.05). The mean METS-IR for participants was 43.44 ± 12.68, with ranges for tertiles 1, 2, and 3 of 17.14–36.75, 36.75–46.82, and 46.82–132.65, respectively. Significant differences were observed among the three METS-IR tertiles in terms of age, sex, race, education level, marital status, poverty-income ratio (PIR), smoking status, hypertension, diabetes, serum cholesterol, and LDL-C levels (all *p* < 0.05). Participants in the higher METS-IR tertiles tended to be male, married or living with a partner, smokers, have high blood pressure, not diagnosed with diabetes, and have higher LDL-C levels compared to those in the lower tertiles (all *p* < 0.05). No significant differences were found in alcohol consumption and physical activity levels (both *p* > 0.05). The baseline characteristics of the participants stratified by METS-IR tertiles are presented in Table 1.

**Table 1:** Baseline characteristics of the study population according to METS-IR tertiles.

Characteristics	Overall	Insulin resistance (METS-IR) index			*p*-value
		Tertile 1 (17.14–36.75)	Tertile 2 (36.75–46.82)	Tertile 3 (46.82–132.65)	
	*n* = 14,794	*n* = 4,931	*n* = 4,931	*n* = 4,932	
Age (years), Mean (SD)	50.01 ± 17.61	47.73 ± 18.95	52.02 ± 17.20	50.27 ± 16.33	<0.001
Sex, (%)					<0.001
Male	7,139 (48.26)	2057 (41.72)	2,611 (52.95)	2,471 (50.10)	
Female	7,655 (51.74)	2,874 (58.28)	2,320 (47.05)	2,461 (49.90)	
Race, (%)					<0.001
Mexican American	2,267 (15.32)	476 (9.65)	858 (17.40)	933 (18.92)	
Other Hispanic	1,628 (11.00)	444 (9.00)	599 (12.15)	585 (11.86)	
Non-Hispanic White	6,112 (41.31)	2,154 (43.68)	1929 (39.12)	2029 (41.14)	
Non-Hispanic Black	2,966 (20.05)	935 (18.96)	983 (19.94)	1,048 (21.25)	
Other Races	1821 (12.31)	922 (18.70)	562 (11.40)	337 (6.83)	
Education level, (%)					<0.001
Less than high school	3,711 (25.11)	993 (20.17)	1,319 (26.77)	1,399 (28.38)	
High school or GED	3,335 (22.56)	1,044 (21.21)	1,133 (23.00)	1,158 (23.49)	
Above high school	7,734 (52.33)	2,886 (58.62)	2,475 (50.23)	2,373 (48.13)	
Marital status, (%)					<0.001
Married or with a partner	8,866 (59.95)	2,773 (56.24)	3,096 (62.80)	2,997 (60.82)	
Single	5,923 (40.05)	2,158 (43.76)	1834 (37.20)	1931 (39.18)	
Smoking status, (%)					<0.001
No	8,232 (55.73)	2,895 (58.73)	2,734 (55.51)	2,603 (52.82)	
Yes	6,550 (44.27)	2034 (41.27)	2,191 (44.49)	2,325 (47.18)	
Drinking status, (%)					0.068
No	3,201 (21.64)	1,027 (20.83)	1,048 (21.25)	1,126 (22.83)	
Yes	8,150 (55.09)	2,773 (56.24)	2,733 (55.42)	2,644 (53.61)	
Unknown	3,443 (23.27)	1,131 (22.94)	1,150 (23.32)	1,162 (23.56)	
Hypertension, (%)					<0.001
No	8,462 (57.20)	3,484 (70.66)	2,751 (55.79)	2,227 (45.15)	
Yes	6,332 (42.80)	1,447 (29.34)	2,180 (44.21)	2,705 (54.85)	
Diabetes, (%)					<0.001
No	11,550 (78.07)	4,494 (91.14)	3,926 (79.62)	3,130 (63.46)	
Yes	3,244 (21.93)	437 (8.86)	1,005 (20.38)	1802 (36.54)	
Physical activities, (%)					0.209
Inactive	9,336 (63.14)	3,124 (63.39)	3,147 (63.85)	3,065 (62.18)	
Vigorous	5,450 (36.86)	1804 (36.61)	1782 (36.15)	1864 (37.82)	
PIR	2.48 ± 1.62	2.61 ± 1.66	2.52 ± 1.62	2.29 ± 1.56	<0.001
Serum cholesterol (mg/dL), Mean (SD)	191.62 ± 41.74	189.88 ± 40.01	193.76 ± 42.20	191.22 ± 42.88	<0.001
Low-density lipoprotein (mg/dL), Mean (SD)	113.25 ± 35.62	108.11 ± 34.23	117.44 ± 36.50	114.25 ± 35.44	<0.001
Fasting glucose (mg/dL), Mean (SD)	110.20 ± 36.65	98.23 ± 17.75	108.51 ± 32.34	123.86 ± 48.34	<0.001
Serum triglycerides (mg/dL), Mean (SD)	125.28 ± 110.38	83.28 ± 42.19	120.31 ± 65.95	172.24 ± 162.56	<0.001
High-density lipoprotein (mg/dL), Mean (SD)	53.90 ± 16.12	65.13 ± 16.67	52.27 ± 12.45	44.30 ± 11.23	<0.001
Body mass index (kg/m^2^), Mean (SD)	29.14 ± 6.93	22.90 ± 2.58	28.26 ± 2.54	36.26 ± 6.39	<0.001
METS-IR, Mean (SD)	43.44 ± 12.68	31.11 ± 3.83	41.60 ± 2.87	57.61 ± 10.14	<0.001
Stroke, %					0.018
No	14,233 (96.21)	4,774 (96.82)	4,736 (96.05)	4,723 (95.76)	
Yes	561 (3.79)	157 (3.18)	195 (3.95)	209 (4.24)	

Among the 14,794 participants, the amount of missing values for the covariates were 14 (0.09%) for education level, 5 (0.03%) for marital status, 12 (0.08%) for smoking status, 3,443 (23.27%) for drinking status, 8 (0.05%) for physical activities, 1,397 (9.44%) for PIR, 243 (1.64%) for low-density lipoprotein. Since “drinking status” as a categorical variable was missing by more than 5%, we treated participants with a missing drinking status variable as a separate category named “unknown”.

### 3.2 METS-IR and the incidence of stroke

We found that higher METS-IR was associated with an increased incidence of stroke. This association was statistically significant in Model 1 (OR = 1.01; 95% CI: 1.00–1.02; *p* = 0.0013) and Model 2 (OR = 1.01; 95% CI: 1.01–1.02; *p* < 0.0001), and it remained consistent in Model 3. Specifically, each unit increase in the METS-IR score was associated with a 1% higher risk of stroke (OR = 1.01; 95% CI: 1.01–1.02; *p* = 0.0006). For sensitivity analyses, we categorized METS-IR as a variable into three tertiles. The results showed that individuals in Tertile 3 had a 31% higher incidence of stroke compared to those in Tertile 1 (OR = 1.31; 95% CI: 1.04–1.63; *p* = 0.0192; Table 2).

**Table 2:** Association between METS-IR and stroke.

METS-IR	Model 1^a^	*p*-value	Model 2^b^	*p*-value	Model 3^c^	*p*-value
Stroke /OR^d^ (95% CI)^e^						
Continues	1.01 (1.00, 1.02)	0.0013	1.01(1.01, 1.02)	<0.0001	1.01 (1.01, 1.02)	0.0006
Categories						
Tertile 1	Reference		Reference		Reference	
Tertile 2	1.25 (1.01, 1.55)	0.0395	1.13 (0.91, 1.41)	0.2736	1.14 (0.91, 1.43)	0.2565
Tertile 3	1.35 (1.09, 1.66)	0.0058	1.40 (1.13, 1.75)	0.0024	1.31(1.04, 1.63)	0.0192
*P* for trend	0.0077		0.0019		0.0185	

In sensitivity analysis, METS-IR was converted from a continuous variable to a categorical variable (tertiles).

^aModel 1: no covariates were adjusted.^

^bModel 2: adjusted for sex, age and race.^

^cModel 3: adjusted for sex, age, race, education level, marital status, family poverty-income ratio, smoking status, drinking status, physical activities, serum cholesterol and low-density lipoprotein cholesterol.^

^dOR: odds ratio.^

^e95% CI: 95% confidence interval.^

**Figure 2:**
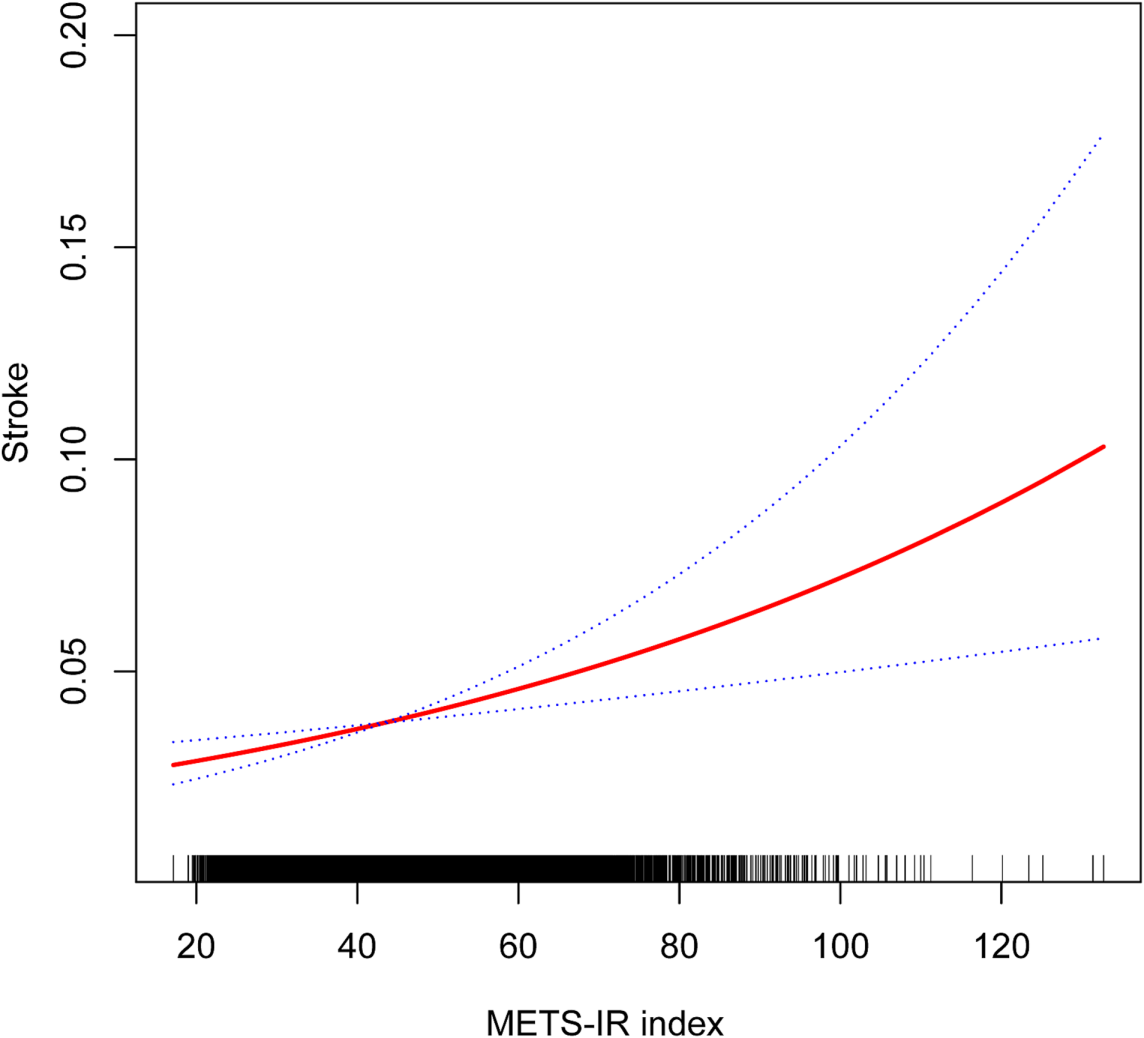
Non-linear associations between METS-IR and stroke.

A non-linear relationship was identified through smooth curve fitting (Figure 2). The solid red line represents the smooth curve fit between variables, while the blue bands illustrate the 95% confidence interval based on the fit. Additionally, we calculated the breakpoint to assess the quantitative relationship between METS-IR and stroke. However, the logarithmic likelihood ratio test yielded a *p*-value >0.05 (Table 3).

**Table 3:** Threshold effect analysis of METS-IR and stroke.

Stroke	Adjusted OR (95% CI)	*p*-value
METS-IR total		
Standard regression model	1.01(1.01, 1.02)	0.0006
Two-piecewise regression model		
Inflection point	58.43	
METS-IR < Inflection point	1.02(1.01, 1.03)	0.0024
METS-IR > Inflection point	1.00(0.98, 1.02)	0.7214
Log-likelihood ratio		0.359

Adjusted for sex, age, race, education level, marital status, family poverty-income ratio, smoking status, drinking status, physical activities, serum cholesterol and low-density lipoprotein.

### 3.3 Subgroup analysis

**Figure 3:**
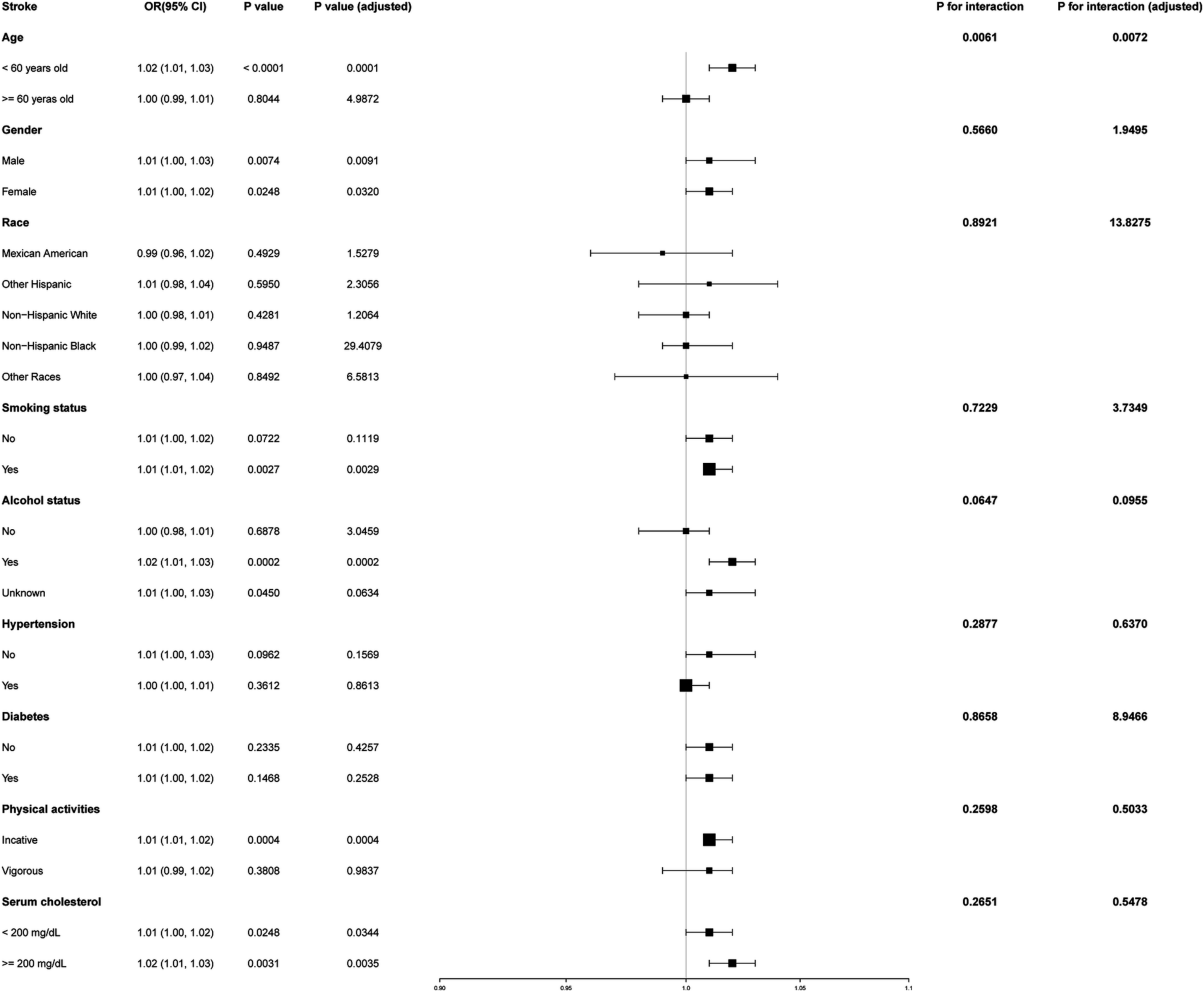
Subgroup analysis of the effect of METS-IR on stroke.

To determine whether the positive association between METS-IR and stroke risk was consistent across various demographic factors, we conducted subgroup analyses and interaction tests stratified by sex, age, race, smoking status, alcohol consumption, hypertension, diabetes, physical activity levels, and serum cholesterol levels. Our results showed that the associations were consistent across all demographic factors, except for age, as illustrated in Figure 3 (all *p* for interaction >0.05). Notably, the association was significantly positive (OR = 1.02; 95% CI: 1.01–1.03; *p* < 0.0001) and more pronounced (*p* for interaction = 0.0061) among individuals under 60 years old.

## 4 Discussion

This study systematically analyzed the association between the Metabolic Score for Insulin Resistance (METS-IR) and stroke incidence in a large, representative sample of the U.S. population. We found a significant positive correlation between METS-IR and the risk of stroke, particularly in individuals under the age of 60. These results not only reinforce the sensitivity and reliability of METS-IR as an indicator of insulin resistance but also offer new insights for early stroke prevention strategies. Given the high prevalence of metabolic syndrome globally and the potential impact of insulin resistance on cerebrovascular health, these findings hold significant clinical relevance. They suggest that managing METS-IR-related parameters, such as fasting plasma glucose, triglycerides, BMI, and HDL-C levels, could effectively reduce the risk of stroke, especially in the context of an aging population and lifestyle changes.

To our knowledge, this is the first study to explore the association between METS-IR and stroke in an American population. METS-IR has been established as a reliable indicator of insulin resistance (13), which is implicated in various metabolism-related diseases such as diabetes (16, 17), visceral adiposity (13), and hyperuricemia (27). Recently, attention has focused on the relationship between METS-IR and cardiovascular disease (28–31). In South Korea, a cohort study involving 17,943 participants found an independent positive association between METS-IR and the prevalence of ischemic heart disease, with 1.9% of participants developing ischemic heart disease over 50 months of follow-up (29). It was also demonstrated that METS-IR, with an optimal threshold of 31.1, is a better predictor of ischemic heart disease than the diagnosis of metabolic syndrome alone (29). A cohort study from China investigated the predictive value of METS-IR for cardiovascular disease and its subtypes in patients with hypertension and obstructive sleep apnea, highlighting its clinical implications for early risk stratification (18). Additionally, it was reported that METS-IR was positively associated with blood pressure in participants with normal BMI, outperforming the TG/HDL-C ratio and TyG index, according to a large cross-sectional study from China (28). Qian et al. found that each quartile increase in METS-IR raised the risk of cardiovascular disease by 38% in middle-aged and elderly Chinese hypertensive patients over the age of 45, with LDL-C acting as a mediator (31). Cai et al. delved into the relationship between METS-IR and stroke within a Chinese population, discovering that higher METS-IR levels are associated with an increased risk of total and ischemic strokes, but not hemorrhagic strokes (19). Using data from previous NHANES cycles, Wang and his colleagues (30) reported a curvilinear relationship between METS-IR and subclinical myocardial injury, which was positively correlated with the incidence of subclinical myocardial injury when METS-IR exceeded the inflection point. More broadly, METS-IR has been extensively used to predict the occurrence (32), severity (32, 33), and prognosis (34) of coronary artery disease. Compared to other insulin resistance indices, including the TG/HDL-C ratio, TyG, and TyG-BMI, METS-IR showed the best predictive value (32, 33). Our results further explore the application of METS-IR in cardiovascular diseases. Elevated METS-IR levels were independently associated with an increased incidence of stroke in Americans, indicating that METS-IR could significantly influence stroke prevalence. Since METS-IR is a readily accessible, affordable, and accurate index, assessing it enables individuals to adopt early lifestyle changes and intervene in risk factors, thus potentially reducing disease incidence, particularly among those under the age of 60.

Neuroinflammation (35), oxidative stress (36), and hemodynamic disorders (37) may explain the link between metabolic syndrome and stroke. Metabolic syndrome is thought to induce a persistent low-grade inflammatory state (38). Chronically inflamed adipose tissue releases pro-inflammatory molecules, including interleukin-1 and tumor necrosis factor-alpha, contributing to systemic inflammation and oxidative stress (2, 39). This disrupts the balance between antioxidant defenses and reactive oxygen species, leading to damage to lipids, proteins, and DNA (40). Additionally, metabolic syndrome has been associated with impaired cerebrovascular reactivity (41). In individuals with metabolic syndrome, the carotid arteries—key suppliers of blood to the central nervous system—become stiffer and exhibit a thicker intima (42). In animal models of metabolic syndrome, researchers have observed brain damage resulting from astrocyte proliferation (43). Astrocytes play crucial roles in neuroinflammation, both protective and detrimental to the central nervous system (44). Their protective functions include maintaining blood–brain barrier integrity, reducing excitotoxicity, releasing neurotrophic factors, and supporting angiogenesis, axonal remodeling, and metabolic processes, all of which aid neurological recovery (44, 45). Additionally, astrocytes help regulate neuroinflammation to localize injury and facilitate vascular repair (46). However, they can also exacerbate neuroinflammation, release toxic factors, and form a glial scar that inhibits axonal regeneration, limiting functional recovery in the chronic phase of stroke (45, 47). Astrocytes may also contribute to excitotoxicity and brain edema by altering ion and neurotransmitter regulation (48). Chronic lipid overload and high glucose levels disrupt astrocyte function, leading to insulin resistance characterized by impaired insulin signaling, reduced glycogen synthesis, and altered gene expression (49). These metabolic disturbances enhance neurotoxicity and promote the secretion of inflammatory cytokines (50). Understanding the interactions between insulin resistance, astrocytic dysfunction, and neuroinflammation could clarify the mechanisms linking METS-IR to stroke, presenting a promising focus for future research.

IR is considered a fundamental cause of metabolic syndrome pathogenesis (11) and is commonly associated with obesity, particularly central or visceral obesity, as well as vascular dysfunction (51, 52). During IR, visceral adipocytes become a major source of circulating inflammatory factors, also known as adipokines (53). The resulting chronic inflammation, endothelial dysfunction, atherosclerosis, and hemodynamic changes are potential mechanisms linking IR to stroke. Chronic inflammation stimulates the pathogenic activity of macrophages and smooth muscle cells, leading to intracellular lipid accumulation and the formation of fatty streaks. These fatty streaks can develop into atherosclerotic lesions as the process progresses (54). In a healthy state, insulin promotes endothelial nitric oxide synthase, which produces nitric oxide, a potent vasodilator, through the PI3K/Akt signaling pathway. However, in the presence of insulin resistance, this pathway is disrupted, reducing nitric oxide production, and resulting in vasoconstriction and vascular endothelial dysfunction (55, 56). Atherosclerosis then develops from a series of pathological events initiated by localized endothelial dysfunction (54). Studies have also shown that insulin resistance enhances platelet activity, aggregation, and adhesion (57–59). These pathological changes alter hemodynamics and narrow the vascular lumen, leading to ischemia and changed tissue nutrient metabolism (60). Previous clinical studies have identified significantly impaired cerebral cortical perfusion in insulin-resistant patients compared to healthy individuals (61).

Conducted on an American population, our study is a large-scale cross-sectional analysis designed to explore the association between METS-IR and stroke. However, several limitations persist. Due to the inherent nature of cross-sectional studies, it is not possible to establish a causal relationship between METS-IR and stroke. Additionally, there is some bias because the outcome variables were collected via questionnaires rather than precise imaging techniques. Moreover, the questionnaire did not differentiate whether the stroke was ischemic or hemorrhagic, thus we were unable to define the association between METS-IR and each subtype of stroke. Furthermore, specific data to directly evaluate certain components of metabolic syndrome, such as visceral adiposity and endothelial dysfunction, were not available in the NHANES database. For example, measurements like abdominal fat assessed by MRI or endothelial function assessed by flow-mediated dilation tests could provide more accurate evaluations of these characteristics, but their absence limited our analysis. Despite these limitations, this cross-sectional study provides new insights into how METS-IR is associated with stroke.

## 5 Conclusion

A positive association between METS-IR and self-reported stroke was observed in an American population. Early intervention in insulin resistance could be an effective strategy for preventing stroke, especially in individuals under 60.

## Data Availability

Publicly available datasets were analyzed in this study. This data can be found at: https://www.cdc.gov/nchs/nhanes/?CDC_AAref_Val=https://www.cdc.gov/nchs/nhanes/index.htm.
